# Novel Method for Loading Microporous Ceramics Bone Grafts by Using a Directional Flow

**DOI:** 10.3390/jfb6041085

**Published:** 2015-12-21

**Authors:** Michael Seidenstuecker, Steffen Kissling, Juergen Ruehe, Norbert P. Suedkamp, Hermann O. Mayr, Anke Bernstein

**Affiliations:** 1 Center for Surgery, Department of Orthopedics and Trauma Surgery, Medical Center-University of Freiburg, Hugstetter str. 55, Freiburg D-79106, Germany; E-Mails: steffenkissling@gmail.com (S.K.); norbert.suedkamp@uniklinik-freiburg.de (N.P.S.); hermann.mayr@uniklinik-freiburg.de (H.O.M.); anke.bernstein@uniklinik-freiburg.de (A.B.); 2 Laboratory for Chemistry and Physics of Interfaces, Department of Microsystems Engineering-IMTEK, University of Freiburg, Georges-Koehler-Allee 103, Freiburg D-79110, Germany; E-Mails: ruehe@imtek.uni-freiburg.de (J.R.)

**Keywords:** β-tricalcium phosphate, porous ceramic, alginate, hydrogel, flow-chamber

## Abstract

The aim of this study was the development of a process for filling the pores of a β-tricalcium phosphate ceramic with interconnected porosity with an alginate hydrogel. For filling of the ceramics, solutions of alginate hydrogel precursors with suitable viscosity were chosen as determined by rheometry*.* For loading of the porous ceramics with the gel the samples were placed at the flow chamber and sealed with silicone seals. By using a vacuum induced directional flow, the samples were loaded with alginate solutions. The loading success was controlled by ESEM and fluorescence imaging using a fluorescent dye (FITC) for staining of the gel. After loading of the pores, the alginate is transformed into a hydrogel through crosslinking with CaCl_2_ solution. The biocompatibility of the obtained composite material was tested with a live dead cell staining by using MG-63 Cells. The loading procedure via vacuum assisted directional flow allowed complete filling of the pores of the ceramics within a few minutes (10 ± 3 min) while loading through simple immersion into the polymer solution or through a conventional vacuum method only gave incomplete filling.

## 1. Introduction

Biomaterials are frequently used for a myriad of purposes in modern clinical practice and in orthopedics, functioning to replace, fix, and stabilize bone after breakage, to restore ligaments and tendons, and for arthroplasty and many other procedures. Previously, non-biodegradable metals such as high-grade stainless steel/surgical steel or titanium alloys [1] were used, as such metals possess good mechanical characteristics, are very biocompatible, and resistant to corrosion. Polymethyl methacrylate (PMMA) bone cement is still used to anchor metallic biomaterials in bone [2]. Due to major advances in tissue engineering, attention has been drawn to bioresorbable materials that are broken down and replaced by the body’s own tissues [3]. Bioresorbable materials serve to deliver bioactive substances that further the healing process in both soft and hard tissue. Biodegradable ceramics are also attracting growing attention [4]. Calcium-phosphate (CaP) bioceramics deserve a special mention here, as they are from a chemical point of view rather similar to the mineral content of bone [5]. The biological response of the body toward these materials also resembles that of native bone during bone regeneration or remodeling. During such naturally occurring reconstruction the bioceramic is broken down and metabolized. In a very similar way as the mineral content in native bone tissue [6]. Bioceramics can be generated synthetically through precipitation reactions or sintering processes and from natural sources, through either allogenic [7] or xenogenic [8] processes. They are currently successfully applied in orthopedic surgery and in dentistry for examples as bone fillers (cements) [9] or granulates [10]. They also serve to coat the shafts of endoprostheses.

Such materials have been in use for a long time as drug carriers for the local release of various bioactive compounds (*i.e.*, antibiotics, analgesics, anti-cancer drugs, hormones, and growth factors) [11]. Active ingredients can be added to the calcium-phosphate cements either in their fluid or solid state [12]. Granulates can be loaded via immersion, during which they are incorporated within the active ingredient's solution for a specific duration [13]. However, a disadvantage of such loading methods is that the active ingredients cannot be released over the course of longer treatment [14]. Similarly, when a ceramic body is employed to which the bioactive compounds are added only through adsorption or through other adhesive processes, also rapid drug release is observed [14,15,16]. During a rapid initial phase most of the bioactive compound is released (“burst release”), making a long term application impossible. Additionally, most active ingredients (e.g., antibiotics and growth factors) are also highly sensitive to high temperatures, so that the active ingredients cannot be added to the mixture while the granulates are being produced [17].

Accordingly, it is necessary to develop a loading method for bioactive porous ceramics, which can take place at ambient temperature and which allows a long-term release of active ingredients. The release of active ingredients from hydrogels (alginate, agarose, chitosan, or gelatins) has been much discussed in the literature [18,19,20], as the slow diffusion of such compounds out of the hydrogels makes a long-term release (even over weeks) possible [21]. To date, however, calcium-phosphate granulates have either been added to the hydrogel [22,23] or the granules have been given an outer coating (a retardant) to prevent early release. Porous ceramic molds have not yet been successfully loaded with hydrogels. Experimental methods such as loading via a centrifuge [24] have proven to be neither practical, nor do they guarantee complete filling. In this study we aimed at loading a resorbable, microporous β-tricalcium phosphate (β-TCP) ceramics having interconnected pores with hydrogels and compare with different methods.

## 2. Results and Discussion

### 2.1. Characterization of the Ceramics

**Figure 1:**
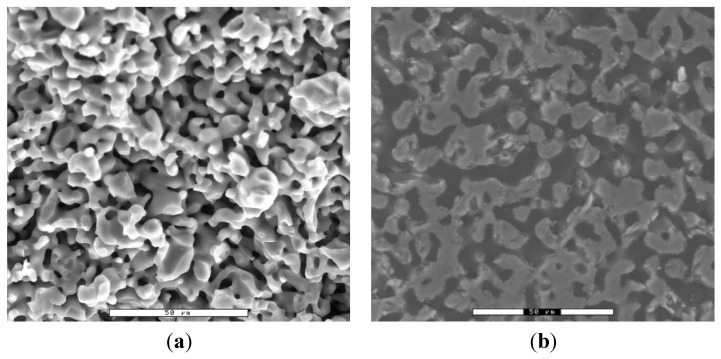
ESEM micrographs of (**a**) a fractured surface of the β-TCP ceramic and; (**b**) a polished section the same ceramics as in (**a**) embedded in Technovit 9100; ESEM images taken with ElectroScan 2020 (ElectroScan, Wilmington, MA, USA) with GSED sensor in WET mode at 0.67 kPa vacuum.

Investigations via ESEM evaluation by image analysis yielded similar results: an overall porosity of 42% and an average pore radius of 2.4 microns. The small variation in the obtained values is probably due to the fact that in the mercury porosimetry measurements the entire ceramic plug was examined, whereas in the ESEM images only the center part of the ceramic plug was analyzed. Figure 1 shows ESEM images of the fractured surface and of a polished section.

**Figure 2:**
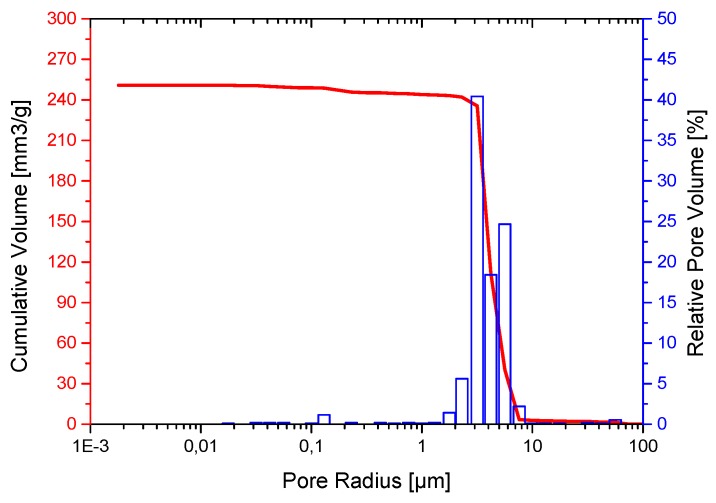
Pore size distribution of β-TCP ceramics obtained by mercury porosimetry.

Figure 2 gives the pore size distribution as obtained by mercury porosimetry. The measurement demonstrates that the ceramic has in addition to the micropores, whose radii are between 1 µm to 10 µm a very small proportion of larger pores with radii 40–70 micron and nanopores with radii between 50 nm to 200 nm [1,2,10]. Mercury porosimetry shows that the microporous β-TCP ceramics have a total porosity of 45% with an average pore radius of 2.8 µm and that the pores are interconnected. Our results determining the median pore size and total porosity in both procedures yielded the same order of magnitude: between 4 µm and 5 µm and 40% and 50% total porosity, respectively. Differences between the two procedures can be attributed to the fact that mercury porosimetry analyzes the entire ceramic element, whereas photo analysis only analyzes a section thereof.

**Figure 3:**
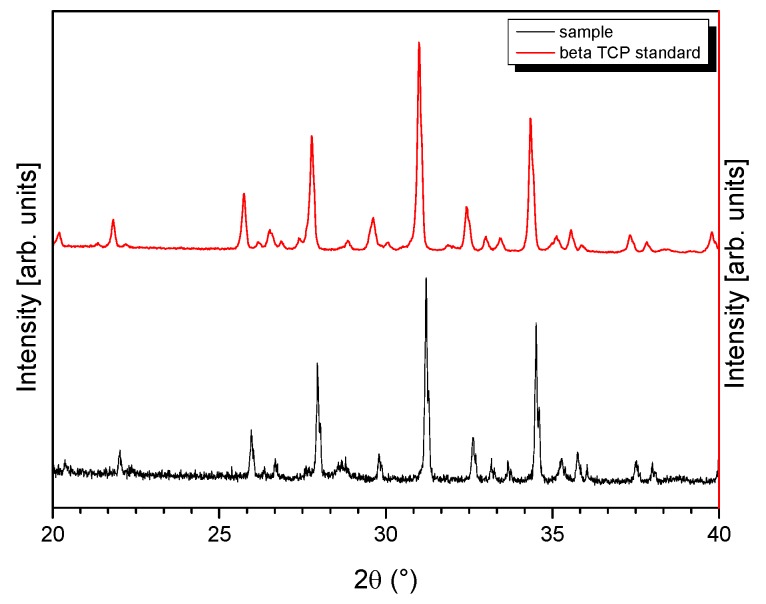
XRD patterns of a standard β-TCP ceramics as depicted in the JCPDS database (red line) and of the prepared β-TCP sample [14].

By EDX Ca, P, and O were detected, characteristic of a calcium phosphate ceramic. Quantification of the EDX spectrum (Ca and P without standards and theoretical *k*-factors) results in a Ca/P ratio of 1.5, which corresponds to β-TCP [17]. Figure 3 shows the XRD pattern of our sample in powder form compared to a β-TCP standard from the JCPDS database (β-TCP = JCPDS 9-169). Comparison of the two spectra reveals a clear consensus with no evidence of a further phase or displacement. The reason for the increased noise in the spectrum of our sample is due to the degree of low milling.

### 2.2. Viscosity Measurements

**Figure 4:**
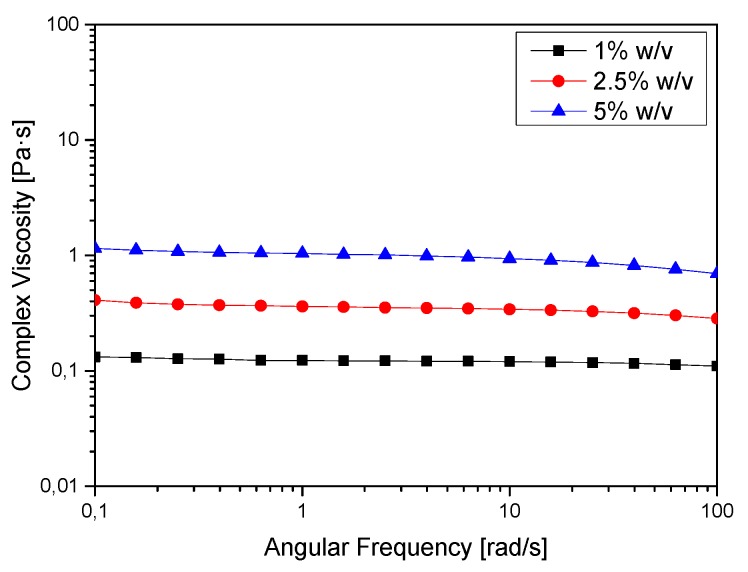
Complex viscosities of alginate sols at concentrations of 1%, 2.5%, and 5% (w/v) as a function of the shear rate.

To allow easy flowing in of the alginate solutions into the pores of the ceramic, the viscosity of the solutions is a decisive parameter. Figure 4 depicts the complex viscosities of the alginate solutions at different concentrations. The higher the concentration of the solution, the higher the level of hydrogel loading of the ceramics, but the more difficult the filling process. In addition, the shear rate varies strongly depending on the size of the pores. Therefore it must be indicated that the viscosity is strongly influenced by the shear [25,26].

### 2.3. Loading Experiments

The charging times were nearly identical in all cases and any differences were associated only with the ceramics’ internal structure. Average loading time was 10 ± 3 min.

**Figure 5:**
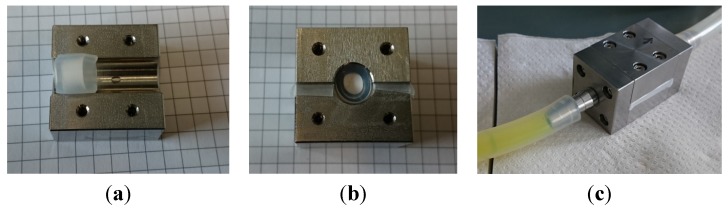
Sealed ceramics inside the flow chamber ; (**a**) top view before closing the chamber, the ceramic is already embedded within a silicone seal; (**b**) frontal view after having screwed down both middle pieces; the intact seals on the left and right between the two halves are readily visible; (**c**) in the left tube, alginate stained with FITC is flown in under ambient pressure, the right transparent tube is attached to a vacuum pump, the seal is visible in the middle between the two halves of the flow chamber.

Simple weighing of the samples prepared by different preparation methods was not helpful to determine the degree of filling as the samples which were obtained by simple immersion or immersion under vacuum contained a rather thick layer surrounding the outer perimeter of the ceramic part. This layer was the result of adhering alginate solution after removal of the sample from solution which dried during processing. Removal of this layer proved to be rather difficult as frequently also material was removed from the outer part of the ceramics. In contrast to this the sample in the flow chamber was sealed rather tightly so that no solution remained after sample withdrawal (see Figure 5).

**Figure 6:**
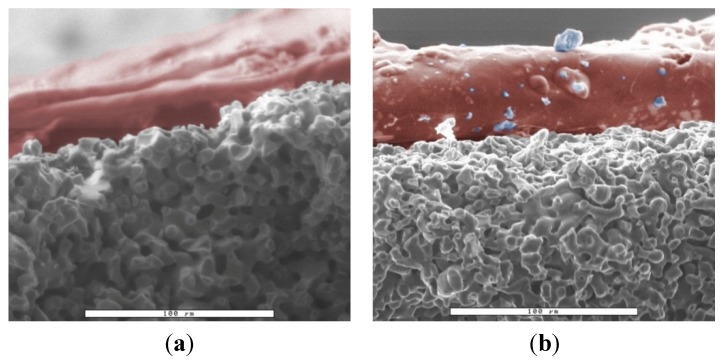
ESEM images of alginate-loaded ceramic; magnification 500×, WET mode at 0.67 mbar; because of the small pore size and the water content of the alginates, only the outer layer of alginate outside the ceramics is visible; for better overview, the image of the alginate layer was colored in red and are ceramic particles on surface (from sample preparation for ESEM) in blue; (**a**) β-TCP loaded with 1% w/v alginate , the outer layer had a thickness of 43 ± 5 µm; (**b**) β-TCP loaded with 2.5% w/v alginate , thin layer had a thickness of 52 ± 5 µm.

As the ESEM images in Figure 6 illustrate, the filled pores are not visible on the ESEM because the pore diameter was only 5 µm, and the evaporation of the water during acquisition of the microscopy images. In these images only the alginic acid remaining on the surface of the ceramic scaffold was visible. For this reason, a second method was used to detect the loaded amount of hydrogel by using a fluorescent dye.

**Figure 7:**
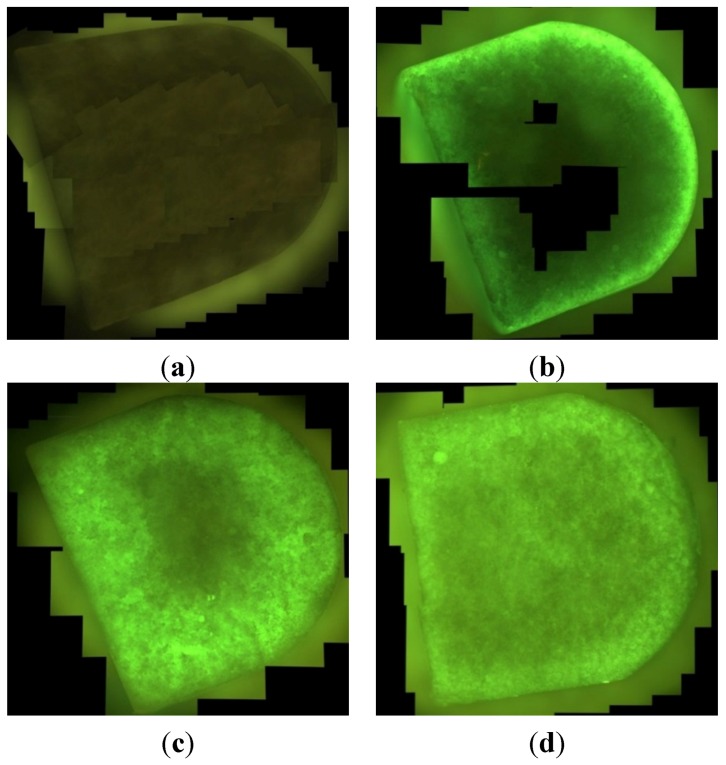
Ceramics filled with different processes: (**a**) unfilled; (**b**) filling via immersion; (**c**) loading via vacuum method and; (**d**) filling via directional flow; the microscopy was repeated for at least three times with ceramics filled with different loading methods; all images taken with Olympus BX 51 fluorescence microscope (Olympus, Shinjuku, Japan) under blue light with λ = 498 nm, sample diameter 7 mm.

In the fluorescence microscopy images the unloaded microporous ceramics revealed a brown color, whereas the FITC-loaded ceramics emitted a light green color (Figure 7). About 40 individual images were taken to construct each panorama image of the fractured surface of the ceramics.

When samples obtained by different loading processes are compared, it becomes obvious that samples filled by using the immersion method, the ceramic became loaded only close to the surface, while the core remains completely unloaded (Figure 7). The penetration depth of the polymer into the ceramics was only *d* = 0.34 ± 0.07 mm resulting in a filling of the pores by only 28% (Table 1). Following the vacuum method a higher load was observed, but again only the periphery is loaded. The vacuum method enabled a penetration down to *d* = 1.82 ± 0.36 mm and a pore filling of 82%. The reason for this incomplete behavior is that the pressure could not be reduced too strongly to avoid freezing of the solution. However, as the sample is completely surrounded by solution, any air still remaining in the porous structure cannot escape and remains in the sample after completion of the filling process [27]. In contrast to this the samples obtained via directional flow in the flow chamber exhibited a very homogeneous fluorescence indicating full loading (100%) (Table 2). Any air still remaining in the sample after application of the vacuum is slowly replaced by the in-flowing aqueous solution. With *p* = 0.05 there were significant differences between the loading methods.

**Table 1:** Penetration depth and loading of the ceramics (7 mm diameter) filled with alginate solutions (2.5 w/v) according to the loading method, samples are analyzed after crosslinking with an external calcium source (*N* = 20).

Loading Method	Penetration Depth (mm)	Loading of the Ceramics (%)
immersion	0.34 ± 0.07	28
vacuum	1.82 ± 0.36	82
directional flow	3.50	100

**Table 2:** Loading times of ceramics as a function of the different alginate concentration and pressure difference applied to the flow chamber (*N* = 10).

Concentration (%w/v)	Loading Time (min)				
	500 mbar	250 mbar	100 mbar	50 mbar	5 mbar
1	42.8 ± 19.0	28.3 ± 16.5	12.1 ± 3,8	5.4 ± 0.3	*
2.5	112.6 ± 14.5	47.0 ± 12.1	25.3 ± 10.5	10.0 ± 3.1	*
5	>120	>120	>120	>120	*

* At this pressure the hydrogel began to freeze within the flow chamber, making loading impossible. After loading process the alginate was crosslinked in 30 mM CaCl_2_ solution

The loading times were studied as a function of the alginate concentration and under pressure applied to the flow chamber. Generally, the higher the pressure difference, the faster the loading was. However, when the pressure was too low (5 mbar) the alginate solution began to freeze inside of the chamber so that no filling could be achieved. The concentration of the solutions was only a very important parameter. When concentrations of 5% were applied the loading process was so slow that within 2 h the loading process was not completed.

### 2.4. Live-Dead Assays

**Figure 8:**
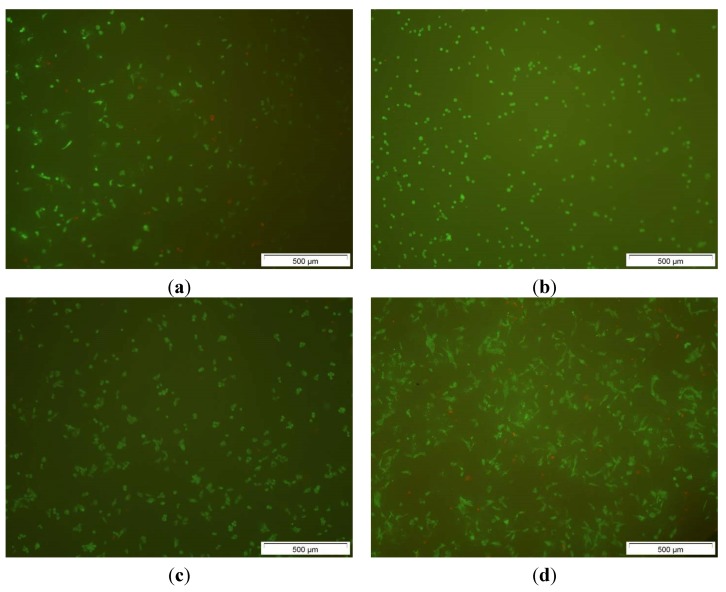
Comparison of live-dead assay at the composite after 24 h of (**a**) a blank β-TCP; (**b**) a composite consisting of β-TCP and alginate; (**c**) the same sample as in (**b**) after 48 h and; (**d**) the sample after 72 h incubation; images taken with 5× magnification on the Olympus BX 51; living cells in green, dead cells in red.

**Figure 9:**
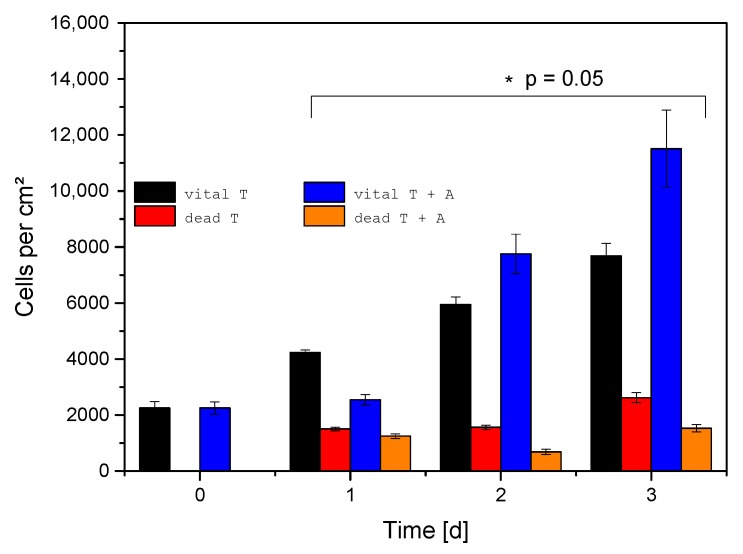
Results from the live-dead assay measurements for empty β-TCP ceramics and β-TCP filled with alginate (*N* = 6); vital T = vital cells on empty β-TCP; vital T + A = vital cells on composite β-TCP + alginate; dead T = dead cells on empty β-TCP; dead T + A = dead cells on composite β-TCP + alginate.

An important question for the application of the generated material is, whether the composite influences viability or proliferation of cells brought into contact with the filled ceramics. To this live-dead assays were carried out after 24 h, 48 h, and 72 h (Figure 8). In addition, experiments on the unfilled ceramics were performed. When the unmodified ceramic and the alginate filled ceramic were compared, it was observed that the number of the MG 63 cells did not change after 24 h (Figure 9). However, it was also evident that the morphology of the cells changed under the influence of alginate from oblong to round (Figure 9), indicating that the cell adhesion to the substrate was significantly decreased. While the cells grow only on the surface of the unmodified ceramic, the cells also penetrate into the gel at the alginate coated samples.

When the cell growth rate on the two samples are compared, it becomes evident that the number of cells on the alginate-loaded β-TCP ceramics is greatly improved compared to that of the unmodified ceramics (Figure 8). The cell numbers increase from 2250 ± 220 viable cells/cm^2^ at the beginning of the cell culture experiments to 2540 ± 190 viable cells/cm^2^ after 24 h on the composite. This is an increase of 13%. After 48 h, 7800 ± 700 viable cells/cm^2^ and after 72 h, 11500 ± 1400 viable cells/cm^2^ were found on the composite. We can thus rule out any negative influence by the presence of the alginate gel onto the cell growth.

## 3. Experimental Section

The microporous ceramic that was chosen to examine is beta tricalcium phosphate (β-TCP) with a total porosity of 40%; the interconnected micropores have an average pore diameter of 5 microns [28,29]. The ceramics were used as cylindrically-shaped bodies 25 mm long and 7 mm in diameter. As a hydrogel precursor, sodium alginate A2158, purchased from Sigma Aldrich (St. Louis, MO, USA) was employed.

### 3.1. Preparation and Characterization of the Ceramics

The ceramic was produced as previously described [14,27]. Briefly, to produce β-TCP plugs, 80 g of α-tricalcium phosphate (α-TCP; Ca_3_(PO_4_)_2_) and 20 g of tricalcium phosphate (Art No 102143, Merck, Switzerland; a mixture of apatite and calcium hydrogen phosphate) were mixed with 60.0 ± 0.20 g of a solution containing 0.2 M Na_2_HPO_4_ (Art. No 4984.2, Carl Roth, Germany) and 1% polyacrylic acid (Art. No 81132, Fluka, Switzerland; Mw = 5.1 kDa). After 2.5 min of intense stirring, the slurry was poured into plastic syringes, whose tip had been cut off (Ø = 23 mm). After 45 minutes, the hardened paste was covered with 10 mL of PBS 7.4 (Gibco DPBS, Thermo Fisher Scientific, Paisley, UK) solution and incubated for three days at 60 °C. The samples (Ø = 23 mm; L ≈ 70 mm) were then dried at the same temperature and sintered at 1250 °C for 4 h. Heating and cooling took place at 1 °C/min. The cylinders were then machined to obtain plugs of 25 mm in length and 10 mm in diameter. The last 2.5 mm of the plugs was given a spherical shape. The samples were then washed in ethanol to remove residual wear particles and calcinated at 900 °C to burn off all organic residues. 

The ceramics were characterized in terms of their porosity and interconnectivity as published previously [14] by means of mercury porosimetry (Porotec PASCAL 140/440, Porotec GmbH, Hofheim, Germany). Moreover, they were embedded in Technovit 9100 (Heraeus Culzer, Wehrheim, Germany) and cut centrally along the longitudinal axis and polished. Electron micrographs were taken using an ESEM (ElectroScan ESEM 2020, Wilmington, MA, USA) in the wet mode at 5 torr with a detector for secondary electrons. The composition of the ceramics was determined by EDS (Oxford INCA, Abingdon, UK) and XRD (Bruker, Billerica, MA, USA). 

### 3.2. Preparation of the Alginate Solutions

Sodium alginate, obtained from Sigma-Aldrich (Art.No A2033, St. Louis, MO, USA), was dissolved in distilled water to give solution with 1%, 2.5%, and 5% w/v concentrations and homogenized for at least 24 h. To enable visualization of the gels by fluorescence microscopy, we added 5 mg/ml FITC (Art.No.3745.2, Carl Roth, Karlsruhe, Germany) to the solutions. To ensure homogeneous distribution of FITC in the alginate solution FITC was added to the distilled water immediately as production started. To prevent bleaching of the dye by ambient light, all solutions were protected by wrapping the containers in aluminum foil.

The alginate solutions (1%, 2.5% and 5% w/v) were measured by using a rheometer (Anton Paar Physica MCR 301, Anton Paar, Graz, Austria) employing the plate-plate method (Ø = 50mm) in the frequency sweep mode. As measurement parameters a deformation γ of 10%, a gap width of 0.4 mm and a frequency range of 0.1–100 rad/s were applied.

### 3.3. Loading of the Ceramics

**Figure 10:**
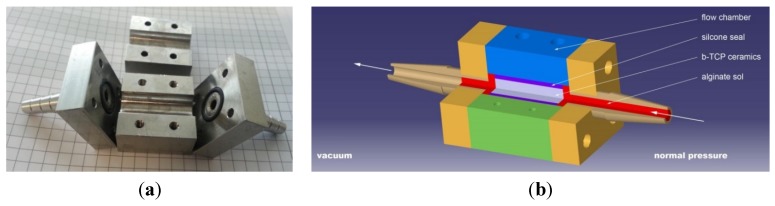
(**a**) Photograph; and (**b**) schematic depiction of the flow chamber used to fill the ceramic samples.

To load the ceramics, a setup was designed and manufactured from stainless steel (X5CrN18-10) (Figure 10). On one side of the flow chamber, a low vacuum (50 mbar) was applied, while atmospheric pressure remained on the other side above the reservoir tank, which contains the polymer solution. Inside of the flow chamber, the ceramic dowels are inserted into a silicone seal (LabMarket, Mannheim, Germany; FDA approved). This seal ensures that loading can take place only through the end surfaces of the microporous ceramic cylinders and that the ceramic cylinder remains immobile during the loading process. To allow visual inspection, transparent vacuum hoses were attached to the chamber. The loading process was stopped when, for at least 1 min, alginate solution exited from the flow chamber. To allow visual inspection transparent tubes were employed for flowing the solution into and out of the chamber. After loading, the ceramics were removed and incubated in 30 mM CaCl_2_ (Art.No. A119.1, Carl Roth, Karlsruhe, Germany). To ensure that no FITC becomes discharged into the calcium solution during cross-linking, FITC was also added to the calcium chloride solution at the same concentration. For comparison purposes, microporous ceramics were loaded by subjecting the ceramics to the alginate solution for 10 min at a vacuum of 50 mbar (“conventional vacuum process”) and by immersion where the ceramic plug was simply placed into the alginate solution under ambient pressure for 24 h. All experiments were carried out with three different concentrations of alginate (1%, 2.5% and 5% w/v) at a temperature of 25°C.

To monitor the loading of the microporous ceramics, the samples were weighed before and after loading with a precision scale (Sartorius BP121S). Each sample was weighed in triplicate and the values then averaged. To obtain electron micrographs of the loaded ceramics, the cylinders were broken along the longitudinal axis and the fracture surface images were taken with an ESEM (Electroscan 2020) with a sensor for secondary electrons at 20 kV. In addition, the samples were imaged via fluorescence microscopy (Olympus BX 51). To obtain a panoramic image, several images were stitched together using the MS Image Composition Editor 1.4.4 (Microsoft, Redmond, WA, USA).

To investigate the dependence of ceramics loading on the hydrogel concentration and applied pressure in the loading process three different concentrations alginate sols were prepared (1%, 2.5% and 5% w/v). To all solutions, FITC was added so that the final concentration was 5 mg/ml. The alginate solutions were loaded into freshly prepared empty TCP ceramics. To study the infusion of the alginate solution into the porous ceramics, the loading process was performed as the pressure at the outlet was reduced to 500 mbar, 250 mbar, 100 mbar, 50 mbar, and 5 mbar at 25°C. The loading times were measured until the alginate sol exits from the flow chambers other side at least 1 min. All experiments were performed at least three times.

### 3.4. Testing of the Biocompatibility of the Ceramic/Gel Composites

To study the influence of the composite onto the behavior of cells in model experiments MG-63 cells (ATCC CRL-1427) were brought into contact with the composite and analyzed via live-dead assay using a live-dead cell staining kit II (Art.No. PK-CA707-30002, Promokine, Germany). Ten composites of porous β-TCP ceramics loaded with alginate under sterile conditions were transferred onto microtiter plates (24-well, flat bottom). MG 63 cell suspensions (2 × 10^4^ cells/mL) and cell culture medium were added to the composites to give a final volume of 1000 µl/well. [28] The cells were cultured for 24 h, 48 h, and 72 h in a humidified atmosphere (37 °C, 5% CO_2_). For staining after the first cultivation, the medium was removed and the cells washed to eliminate serum esterase activity. Subsequently, the cells were stained according to a standard protocol [30] and inspected by fluorescence microscopy. Living cells emit a green fluoresence and dead cells a red fluorescence when inspected under blue light.

### 3.5. Statistics

Data are expressed as mean values ± standard deviation of the mean and analyzed by one-way analysis of variance (ANOVA). The level of statistical significance was set as *p* < 0.05. For statistical calculations Origin 9.1 Professional SR1 (OriginLab) was used.

## 4. Conclusions

In this study we introduced a new method for loading of a porous β-TCP ceramics designed for bone replacement with alginate hydrogel precursors by placing the sample into a vacuum and using a directional flow to fill the pores of the ceramics with the alginate solution. We could show that the loading of the microporous ceramics with a hydrogel precursor using a combination of air removal from the porous material and directional flow into the sample could be a good alternative to conventional methods such as simple immersion or vacuum assisted filling. The flow assisted impregnation of the ceramics proceeds much faster while at the same time the total loading of the porous ceramics is increased from roughly 28% (immersion) or 82% (conventional vacuum method) to 100% and complete filling of the porous ceramic was observed. No negative effect of the alginate filling of the ceramics on the cell growth was found. In conclusion, the developed loading method is therefore well-suited for impregnating porous ceramics with hydrogel precursors. In a follow-up paper we describe how the calcium induced crosslinking occurs and how antibiotics are released from the hydrogel filled ceramics.
